# Fast and selective room-temperature hydrogen sensing of oxygen-deficient orthorhombic Nb_2_O_5_ nanobelts†

**DOI:** 10.1039/d4ra08878f

**Published:** 2025-04-22

**Authors:** Piaoyun Yang, Qinyuan Gao, Yijing Fan, Chunya Luo, Sha Li, Yanan Zou, Xianghui Zhang, Haoshuang Gu, Zhao Wang

**Affiliations:** a Hubei Expert Workstation of Terahertz Technology and Advanced Energy Materials & Devices, College of Physics and Electromechanical Engineering, Hubei University of Education Wuhan 430205 P.R. China yangpiaoyun@hue.edu.cn; b Hubei Key Laboratory of Micro/Nano-Electronic Materials and Devices, School of Microelectronics, Hubei University Wuhan 430062 P.R. China xhzhang@hubu.edu.cn; c College of Science, Jilin Institute of Chemical Technology Jilin 132000 P.R. China zouyanan@jlict.edu.cn

## Abstract

The increasing demand for hydrogen as a clean and renewable energy source necessitates the development of efficient and reliable hydrogen sensing technologies. This study presents the preparation of oxygen-deficient orthorhombic Nb_2_O_5_ nanobelts for room-temperature chemiresistive hydrogen sensing. The nanobelts were synthesized by converting the H_3_ONb_3_O_8_ nanobelts into orthorhombic Nb_2_O_5_ through a calcination-based topochemical transformation process. The content of oxygen vacancy defects in the nanobelts was effectively modified by post-annealing treatments, without introducing undesirable phase transition. The results revealed that the hydrogen sensing performance of Nb_2_O_5_ nanobelts is closely linked to the oxygen vacancy content. With optimal defect concentration, the proposed chemiresistive sensors demonstrated significantly enhanced room-temperature hydrogen response, achieving a sensor response of 10.3 and response time down to 28 s, to 5000 ppm hydrogen. The sensor also exhibited good selectivity against various interference gases, highlighting its great potential for fast and accurate hydrogen leak detection in practical applications.

## Abstract 2

Fast, sensitive and selective room-temperature hydrogen sensing is achieved by utilizing oxygen-deficient orthorhombic Nb_2_O_5_ nanobelts.

## Graphical abstract

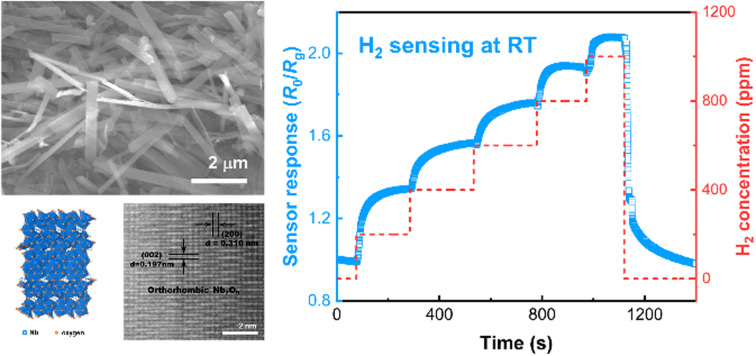

## Introduction

Chemiresistive hydrogen sensors based on semiconductor oxides have attracted significant attention for their potential in detecting hydrogen leakage,^1,2^ which is critical for the safe handling of explosive hydrogen gas as a clean and renewable energy carrier.^3^ Traditional semiconductor sensors, composed of hydrogen-sensitive oxides such as SnO_2_, ZnO and TiO_2_, typically operate at elevated temperatures (above 200 °C) to achieve optimal sensing performance.^4–6^ However, this requirement raises several critical issues, including high power consumption, susceptibility to cross-sensitivity with other interfering gases, and sensor poisoning.^7–9^ Therefore, the development of novel sensing materials and devices that enable fast, sensitive and selective hydrogen gas sensing at reduced working temperatures, particularly at room temperature, is essential for practical applications.^10,11^

Niobium pentoxides (Nb_2_O_5_), a representative wide-band-gap semiconductor oxide,^12,13^ has emerged as a promising candidate for room-temperature hydrogen sensing due to its exceptional catalytic capabilities for hydrogen dissociation,^14–16^ good chemical resistance and tunable electronic properties.^17^ Orthorhombic Nb_2_O_5_, in particular, has demonstrated significant potential for high-performance hydrogen sensing owing to its unique crystal structure, which promotes the formation and migration of oxygen vacancies.^18–20^ These oxygen defects play a pivotal role in modulating the electronic properties of the materials, thereby influencing its gas–solid interaction with the target gas molecules, such as hydrogen.^21,22^ Consequently, the ability to control and optimise oxygen vacancy defects in orthorhombic Nb_2_O_5_ nanostructures could thus significantly enhance their hydrogen sensing performance, especially at room temperature.

However, the polymorphic nature of Nb_2_O_5_ presents challenges in the effective modulation of oxygen vacancies.^23^ Although the orthorhombic phase is generally considered the most stable phase of Nb_2_O_5_ at ambient conditions, it can undergo phase transitions under specific conditions, particularly when lattice oxygen is removed.^24^ The loss of lattice oxygens results in increased lattice distortion, driving the system towards a more energetically favourable state, *e.g.* the monoclinic Nb_2_O_5-*x*_ phase,^25,26^ which exhibit different arrangement of NbO_6_ octahedra. Thus, a significant challenge in developing Nb_2_O_5_-based hydrogen sensing materials lies in the precise control of oxygen vacancy content while preventing the onset of undesired phase transitions that could degrade the sensing performance.

In this work, we present the preparation of orthorhombic Nb_2_O_5_ nanobelts using hydrothermally synthesized KNb_3_O_8_ nanobelts as the intermediate products in a topochemical transformation process. By employing a two-step annealing strategy, we obtain a pure phase of orthorhombic Nb_2_O_5_ nanobelts with adjustable oxygen vacancy content. The formation of oxygen vacancies significantly enhances the room-temperature hydrogen sensing performance of the orthorhombic Nb_2_O_5_ nanobelts. Under optimal defect concentrations, we achieve fast, sensitive and selective hydrogen sensing at room temperature.

## Experimental details

### Materials preparation and characterization

Nb_2_O_5_ nanobelts were prepared through a combined topochemical approach, similar to the procedures reported in our previous work.^19^ Initially, analytical grade potassium hydroxide (52.8 mg) and metallic niobium powder (93.6 mg), both sourced from Sinopharm-China, served as the precursors for the hydrothermal synthesis of KNb_3_O_8_ nanobelts. The hydrothermal reaction was conducted at 200 °C for 24 h. Following natural cooling to ambient temperature, the resultant sample was extracted from the autoclave and subjected to centrifugation washing to isolate the desired KNb_3_O_8_ nanobelts. These intermediate products were subsequently immersed in a 2 M HCl solution for an ion-exchange reaction, which proceeded for 24 h, yielding H_3_ONb_3_O_8_ nanobelts. The resultant white powder, comprising H_3_ONb_3_O_8_ nanobelts, was then calcined in air at 800 °C for 30 min using a rapid thermal processing (RTP) furnace with a heating rate of 20 °C s^−1^. Upon cooling, the orthorhombic Nb_2_O_5_ nanobelts, constituting the final product, were retrieved from the furnace.

The surface morphologies of the products were examined using a field emission scanning electron microscope (SEM, JEOL JSM-7100F). The crystal structure of the samples was characterized *via* X-ray diffraction (XRD, Bruker D8A, Cu Kα, *λ* = 0.15406 nm). The microstructure of the nanobelts was elucidated through high-angle annular dark-field (HAADF) imaging in the scanning transmission electron microscopy (STEM) mode of a transmission electron microscope (TEM, FEI Tecnai G2). The valence states of the products were analyzed using X-ray photoelectron spectroscopy (XPS, ThermoFisher Scientific Escalab-250Xi).

### Sensor fabrication and testing

Chemiresistive hydrogen gas sensors were constructed using pre-patterned Pt/Ti interdigital electrodes (IDEs) on quartz glass substrates. The IDEs were deposited using DC Sputtering, with an interspacing of 100 μm. As illustrated in Fig. S1(a),† the fabrication process involved dip-coating 10 μL of the synthesized Nb_2_O_5_ nanobelts (dispersed in DI water, 20 mg mL^−1^) onto the sensing area of the IDEs, followed by a post-annealing (PA) treatment in an argon atmosphere at 500 °C. The hydrogen sensing performance of these sensors was evaluated using a custom-designed gas sensor testing system. Within this system, the target gas was generated in the testing chamber (with a volume of 500 mL) by regulating the injection volume of hydrogen gas with a mass flow controller (flow rate of 10 sccm) and the computer-controlled electromagnetic valves, as illustrated in Fig. S1(b).† The sensor signal was monitored using a Keithley 2400 sourcemeter. After approaching response saturation, the hydrogen gas was exhausted from the testing chamber using dry air to test the recovery behavior of the sensor. The sensor response was defined as the ratio of the baseline resistance (*R*_0_) in air to the resistance (*R*_g_) in the presence of hydrogen gas. The response time was defined as the duration required for the sensor to achieve 90% of the total signal change upon exposure to the target gas.

## Results and discussion

**Fig. 1:**
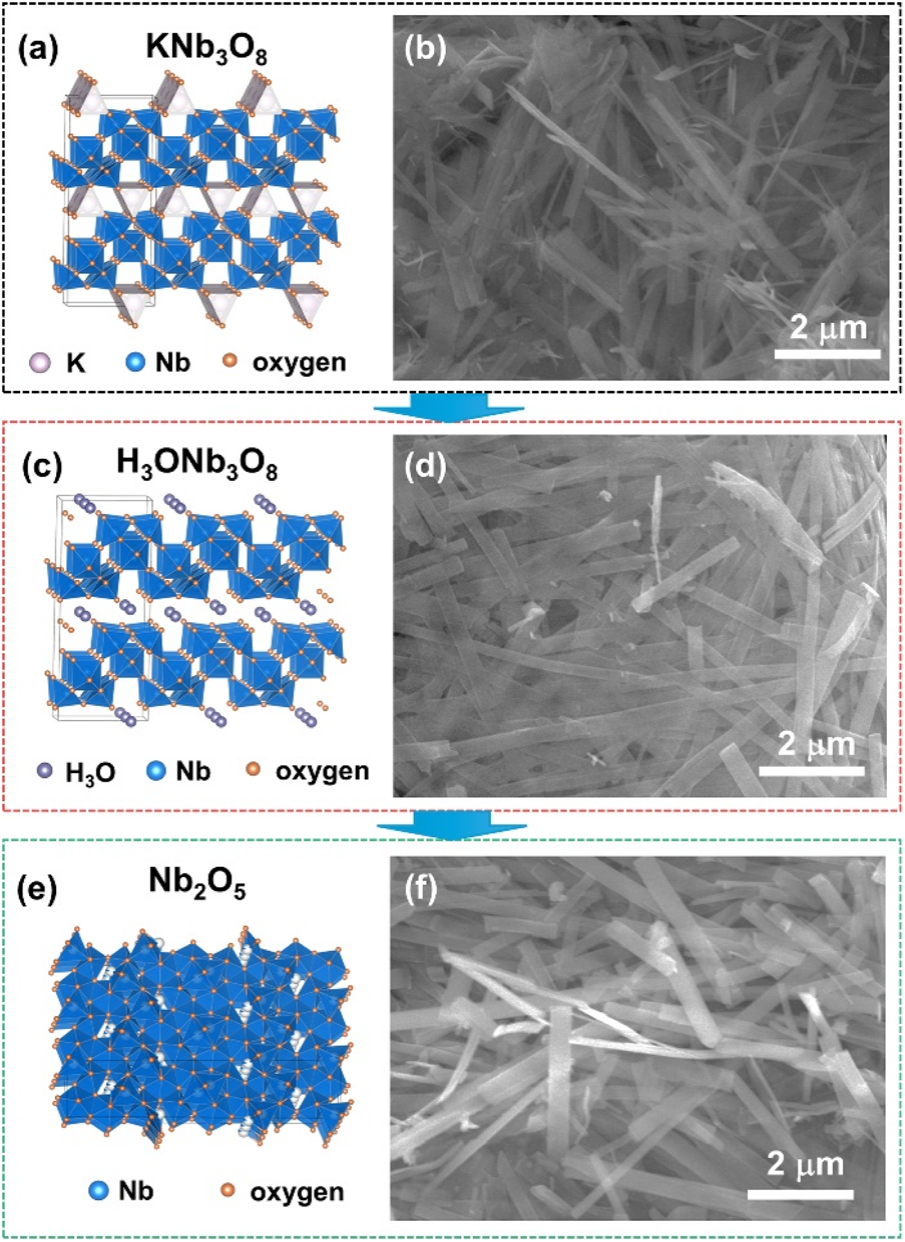
Schematic lattice structure and SEM images of the intermediate and final product during the preparation of Nb_2_O_5_ nanobelts. (a and b) Intermediate KNb_3_O_8_ nanobelts. (c and d) Intermediate H_3_ONb_3_O_8_ nanobelts. (e and f) Orthorhombic Nb_2_O_5_ nanobelts.

Fig. 1 presents the schematic lattice structures and SEM images of the intermediate and final products obtained during the synthesis process. Initially, KNb_3_O_8_ (Fig. 1(a and b)) were synthesized *via* a conventional hydrothermal method, exhibiting belt-like ultrathin one-dimensional (1D) nanostructures. This morphology arises from their layered lattice structure, where Nb–O layers are stacked and interconnected by potassium ions. Following an ion-exchange process in HCl solution, the KNb_3_O_8_ nanobelts were transformed into H_3_ONb_3_O_8_ nanobelts (Fig. 1(c and d)), as K^+^ ions were replaced by H_3_O^+^ ions. These intermediate products underwent calcination in air at 800 °C, resulting in the topochemical transformation into orthorhombic Nb_2_O_5_ nanobelts (Fig. 1(e and f)). The resulting Nb_2_O_5_ nanobelts retained the 1D nanostructure of the intermediates, with dimensions ranging from approximately 100 to 250 nm in width and 40 to 100 nm in thickness.

**Fig. 2:**
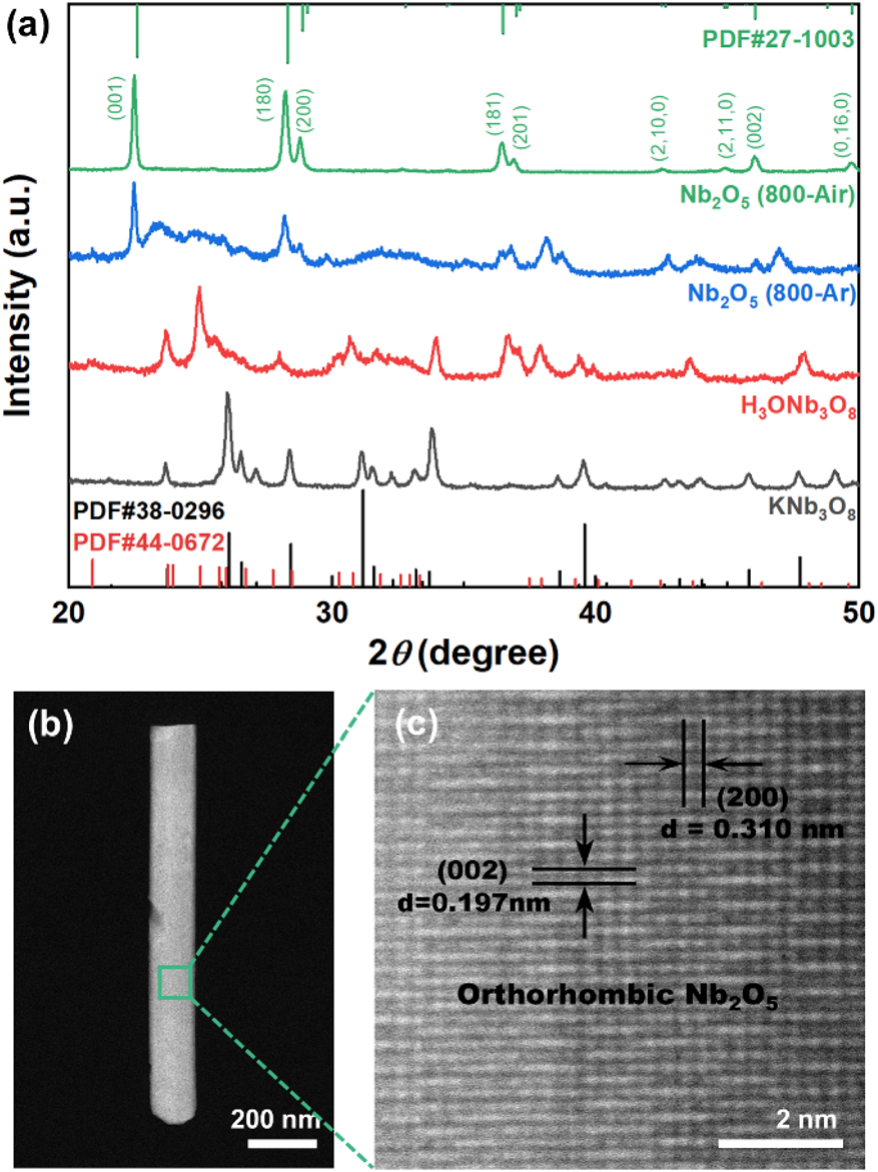
The XRD and TEM characterization results of the Nb_2_O_5_ nanobelts. (a) XRD curves of as-prepared Nb_2_O_5_ nanobelts, comparing with the final samples obtained by calcination in Ar atmosphere at 800 °C and the intermediate products. (b and c) HAADF and HRTEM images of an individual orthorhombic Nb_2_O_5_ nanobelt obtained after the calcination in air at 800 °C.

Fig. 2(a) displays the XRD patterns of both the intermediate and final Nb_2_O_5_ products. The data confirm the formation of KNb_3_O_8_ (JCPDS cards No. 38-0296) and H_3_ONb_3_O_8_ (No. 44-0672) following the hydrothermal and ion-exchange processes, respectively. The XRD pattern of the final product (Nb_2_O_5_ 800-Air) aligns well with the standard orthorhombic Nb_2_O_5_ (JCPDS Card No. 27-1003), with no diffraction peaks from intermediate phases, confirming the purity of the orthorhombic Nb_2_O_5_ nanobelts. Samples calcined in an argon atmosphere (denoted as Nb_2_O_5_ 800-Ar) were also analysed. These samples exhibited additional impurity peaks corresponding to lower-valence niobium oxides, attributed to excessive oxygen loss during calcination in a reducing argon environment.^23^

Fig. 2(b and c) provides TEM characterization of the as-prepared Nb_2_O_5_ nanobelt. High-resolution HAADF-STEM imaging confirmed that the nanobelts' axial direction aligns with the [001] orientation of the orthorhombic Nb_2_O_5_ lattice, while the thickness direction aligns with the [010] orientation. Unlike the polycrystalline Nb_2_O_5_ nanorods previously reported,^19^ no grain boundaries were observed in the nanobelts, indicating their single-crystal nature. This single-crystal structure, due to the ultrathin belt-like morphology, is advantageous for enhancing the hydrogen sensitivity of the sensor layer.^27^

**Fig. 3:**
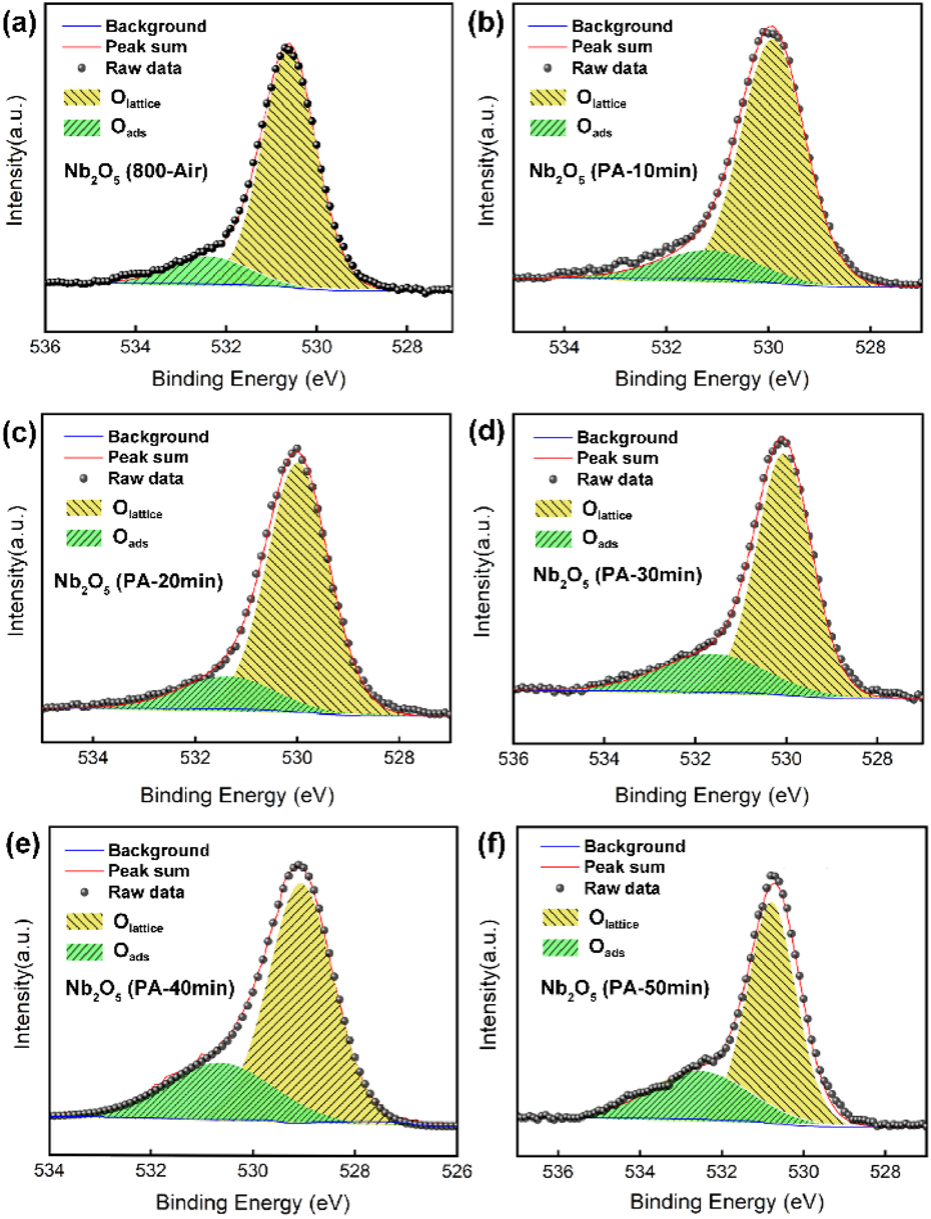
The O 1s XPS spectra of (a) as-prepared Nb_2_O_5_ nanobelts and (b–f) samples obtained after post-annealing in Ar at 500 °C for (b) 10 min, (c) 20 min, (d) 30 min, (e) 40 min, and (f) 50 min.

To modify the oxygen vacancy defects in the as-prepared Nb_2_O_5_ nanobelts, pristine samples (Nb_2_O_5_ 800-Air) underwent further annealing in an argon atmosphere at 500 °C for varying post-annealing (PA) durations (denoted as PA-10 min to PA-50 min). XPS O 1s spectra were analysed to assess the relative content of oxygen defects. Fig. 3(a) shows two distinct peaks at binding energies of 530.6 eV and 532.4 eV, corresponding to lattice oxygen (O_lattice_) and adsorbed oxygen (O_ads_), respectively. Initially, the relative content of O_ads_ was approximately 14.4%. After 10 minutes of PA, this content increased to 16.7%, and further rose to 19.4%, 23.7%, 29.5%, and 30.9% as the PA time extended to 20, 30, 40, and 50 minutes, respectively. Previous studies have linked the presence of O_ads_ to the formation of oxygen vacancy defects in orthorhombic Nb_2_O_5_ nanomaterials, according to the electron paramagnetic resonance spectra.^19,28^ Thus, the increase in O_ads_ content indicates a corresponding increase in oxygen vacancies. XRD patterns of the post-annealed samples (Fig. S2†) confirmed that no phase transitions occurred following the PA treatment.

**Fig. 4:**
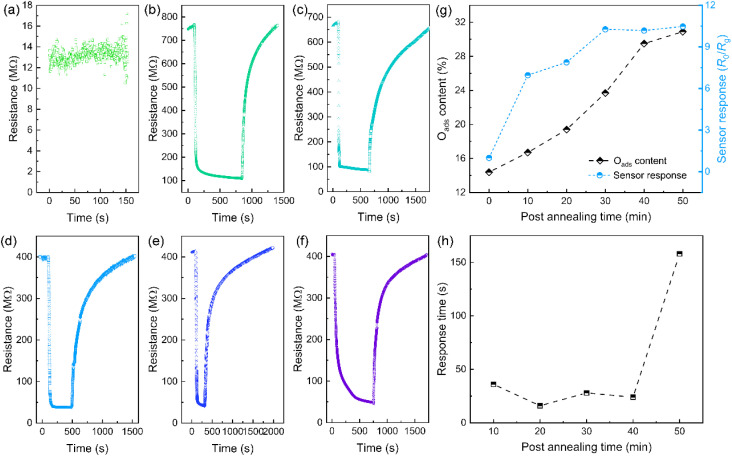
The room-temperature hydrogen response of the chemiresistive sensors based on the orthorhombic Nb_2_O_5_ nanobelts treated by post annealing for different durations. (a) Before post annealing; (b) 10 min; (c) 20 min; (d) 30 min; (e) 40 min; (f) 50 min; (g) variation of O_ads_ content and sensor response with the post annealing time; (h) variation of sensor response time with the post annealing time.

Chemiresistive gas sensors were fabricated by dip-coating the as-prepared Nb_2_O_5_ nanobelts (prior to PA) onto IDEs on a quartz glass substrate, followed by post-annealing in an Ar atmosphere at 500 °C. All devices show linear *I–V* characteristic in air at room temperature (Fig. S3†), indicating the formation of Ohmic contact between the sensing layer and the electrodes. Fig. 4 illustrates the dynamic sensor response (*R*_0_/*R*_g_) to 5000 ppm hydrogen for sensors before and after PA treatment, measured in air at room temperature. Sensors without PA treatment exhibited no response to hydrogen at room whereas post-annealed samples demonstrated high sensitivity, with a full sensor response exceeding 7. Sensor response significantly improved with increased annealing time from 10 to 30 minutes, reaching 10.3 at 30 min, with a response time of 28 s. However, further extending the annealing time to 50 minutes resulted in only slight fluctuations in sensor response and considerably slowed response rates. Fig. 4(b), shows that the enhancement in sensor response correlates well with the increasing O_ads_ content in post-annealed Nb_2_O_5_ nanobelts for annealing times below 40 minutes, indicating that performance optimization is closely tied to oxygen vacancy formation. However, prolonged post-annealing led to nanobelt rupture and recrystallization (Fig. S4†), increasing polycrystalline interfaces in the sensing layers that constrained charge conduction and diffusion efficiency, thereby reducing room-temperature hydrogen sensitivity and response rate.^29^

**Fig. 5:**
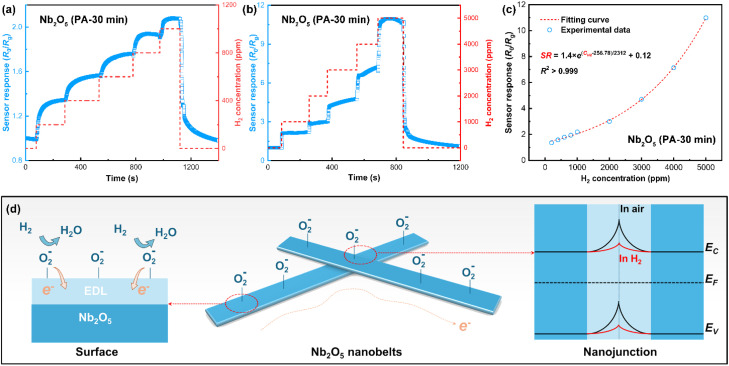
The room-temperature response of the chemiresistive sensors based on Nb_2_O_5_ nanobelts (PA-30 min) to hydrogen gas with varying concentrations in air. (a and b) Continuous sensor response to hydrogen with concentration increasing from (a) 200 to 1000 ppm, and (b) 1000 to 5000 ppm. (c) The relationship curve between the full sensor response and hydrogen concentration. (d) Schematic illustrations for the hydrogen sensing mechanism of the sensing layer based on interlacing Nb_2_O_5_ nanobelts.

Considering the optimal room-temperature hydrogen sensitivity and response time of the Nb_2_O_5_ PA-30 min sample, a detailed analysis of its hydrogen sensing performance was conducted. Fig. 5(a and b) shows the sensor response to hydrogen concentrations increasing from 200 to 5000 ppm, simulating continuous response characteristics during hydrogen leakage and accumulation. Results in Fig. 5(c) reveal that the full sensor response increases exponentially with hydrogen concentration. The experimental data fit well to an exponential function with *R*^2^ > 0.999, facilitating fast and straightforward signal processing for accurate hydrogen detection. As illustrated in Fig. 5(d), pre-adsorbed oxygens (O_2^−^_) on the Nb_2_O_5_ nanobelts' surface (preferentially at oxygen vacancy sites) reacts with the physiosorbed hydrogen molecules, releasing trapped electrons and reducing electron depletion layers (EDLs) near the surface.^30^ As the sensing layers comprise numerous Nb_2_O_5_ nanobelts assembled on the substrate, electron transport at room temperature is predominantly governed by thermionic emission across nanojunctions between adjacent nanobelts, with current density exponentially related to interface barrier height (*J* ∝ exp(−*qϕ*/*k*_B_*T*).^31^ It has been demonstrated that diminished EDLs lower the interface potential barrier height at nanojunctions.^27^ Consequently, the sensor response *R*_0_/*R*_g_ is exponentially related to potential barrier height variation, interpreted as *R*_0_/*R*_g_ = *J*_g_/*J*_0_ ∝ exp (*q*Δ*ϕ*/*k*_B_*T*), where Δ*ϕ* = *ϕ*_0_ − *ϕ*_g_. Given the ppm-level hydrogen concentration, the number of hydrogen atoms participating in surface reactions is much lower than the O_ads_ content. As a result, barrier height variation is linearly related to the reactant amount (*i.e.*, hydrogen gas concentration).^27^ Thus, the sensor response theoretically exhibits an exponential relationship with increasing hydrogen concentration rather than the classical Langmuir and Power-law models (fitting results shown in Fig. S5†) that typically applicable to surface adsorption-limited mechanisms, which is in consistency with the experimental results shown in Fig. 5(c).

**Fig. 6:**
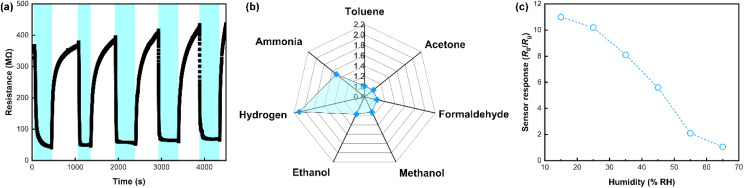
Detailed hydrogen sensing performance of the chemiresistive sensor based on Nb_2_O_5_ PA-30 min. (a) Repeated response to 4000 ppm hydrogen within 5 continuous response and recovery cycles. (b) Radar chart showing the sensor response to different analyte gases, each at a concentration of 1000 ppm and measured at room temperature with dry air as the background atmosphere. (c) Variation of sensor response with the increase in relative humidity.

Table 1 summarizes key sensor parameters, including sensor response, response time, and operating temperature for Nb_2_O_5_-based hydrogen sensors from this study and previous literature. The sensor proposed herein, based on orthorhombic Nb_2_O_5_ nanobelts with modified oxygen vacancies, demonstrates significant superiority in these key indicators. Furthermore, as shown in Fig. 6(a), the sensor exhibited good repeatability over five continuous response and recovery cycles. The full sensor response remained between 6.8 and 7.3 after the first cycle, showing ∼6.8% fluctuations, possibly due to gas distribution errors. It also showed good reproducibility with a deviation of sensor response <±10% among 4 different samples, as shown in Fig. S6.† Additionally, the sensor displayed outstanding hydrogen selectivity against interference gases such as NH_3_, ethanol, methanol, formaldehyde, acetone and toluene, as shown in Fig. 6(b). The post-annealing process introduces abundant oxygen vacancies and surface-adsorbed O_2^−^_ species, which create localized electric fields and defect sites preferentially interacting with hydrogen. Hydrogen molecules exhibit strong physisorption *via* van der Waals forces at these defective sites due to their small molecular size (0.289 nm) and molecular weight (∼2.016 g mol^−1^), which facilitating significantly faster diffusion rate at room temperature than that of ammonia, ethanol and other larger gas molecules. Moreover, gases like NH_3_ and C_2_H_5_OH undergo more complex reaction pathways than hydrogen with the O_2^−^_ species, which demands higher activation energy and leads to slower electron release.^35,36^ Therefore, the outstanding hydrogen selectivity of the Nb_2_O_5_ nanobelts after PA treatment can be attributed to the synergistic mechanisms related to the selective physisorption of hydrogen molecules and their redox specificity driven by the O_2^−^_ species. However, due to the adsorbed-oxygen-mediated hydrogen sensing mechanism, the sensor's performance varied with increasing relative humidity in the testing chamber. As shown in Fig. 6(c), the full sensor response to 5000 ppm hydrogen decreased from ∼11 to 1.05 as relative humidity increased from 15% RH to 65% RH, likely due to water molecules passivating surface reactive sites (adsorbed oxygen), indicating the need for further calibration, such as signal processing, for practical applications.

**Table 1:** Comparation on the key sensor parameters of the as-prepared samples with the reported Nb_2_O_5_-based sensors

Materials	Sensor response (*R*_0_/*R*_g_)	Response time (s)	Working temperature (^o^C)
Nanoporous Nb_2_O_5_ films^18^	1.33 (5000 ppm)	260	100
Tetragonal Nb_2_O_5_ nanowires^17^	44 (2000 ppm)	100	25 °C
Nb_2_O_5_/ZnO nanorods^32^	3.13 (5000 ppm)	24	300 °C
Monoclinic Nb_2_O_5_ nanorods^32^	1.38 (1000 ppm)	29	300 °C
NiO–Nb_2_O_5_ nanoparticles^33^	2.23 (1000 ppm)	85	25 °C
Hexagonal Nb_2_O_5_ nanorods^34^	1.89 (1000 ppm)	70	25 °C
Orthorhombic Nb_2_O_5_ nanobelts^28^	1.32 (1000 ppm)^a^	28	25 °C
Orthorhombic Nb_2_O_5_ nanorods^19^	1.37 (1000 ppm)	125	25 °C
Orthorhombic Nb_2_O_5_ nanobelts (this work)	2.17 (1000 ppm) 10.3 (5000 ppm)	28	25 °C

a P-type response, for which the sensor response is defined as *R*_g_/*R*_0_.

Additionally, the device also exhibited temperature dependent sensor performance, wherein the sensor response to 5000 ppm hydrogen in air decreased from 10.2 at 25 °C to 2.84 at 75 °C, and further dropped to near-zero response (noise level) at 100 °C (Fig. S7†). The drop in sensor response under raised temperature can be attributed to the passivation of oxygen vacancies and the reduced contribution of interface barrier height at higher temperatures. The result indicates the effective working temperature range from room temperature to 75 °C, which can meet the hydrogen detection requirements in conventional atmospheric environments, especially in open environments such as hydrogen refuelling stations. Moreover, it should be noted that the oxygen vacancies may also be passivated during long-term storage in ambient conditions. A drop in sensor response was found when the sensors were storage for over 4 weeks (Fig. S8†), suggesting that a optimized storage condition is necessary for maintaining the sensing activity of the as-prepared samples.

## Conclusions

In this work, orthorhombic Nb_2_O_5_ nanobelts were prepared using a topochemical transformation process. The hydrothermally synthesized KNb_3_O_8_ nanobelts were transformed into H_3_ONb_3_O_8_ through an ion-exchange process, which were then converted into orthorhombic Nb_2_O_5_ after a calcination treatment in air at 800 °C, maintaining the belt-like 1D nanostructure of the starting KNb_3_O_8_ materials. By employing a post-annealing treatment at 500 °C in argon atmosphere, the relative content of pre-adsorbed oxygens can be effectively increased from 14.4% to 30.9%, which can be attributed to the formation of oxygen vacancy defects due to the loss of lattice oxygens during annealing. The as-prepared Nb_2_O_5_ nanobelts exhibited significantly enhance room-temperature hydrogen sensing performance after the introduction of oxygen vacancy defects. The proposed chemiresistive sensors based on the nanobelts with post-annealing time of 30 min exhibit optimal sensor performance, with sensor response up to 10.3 and the response time down to 28 s. The sensor response exhibited typical exponential relation with the increasing concentration of hydrogen, consistent with the thermionic-emission-dominated electron transport in the polycrystalline sensing layer composed of agglomerated nanobelts. The proposed sensor also shows good repeatability and hydrogen selectivity against other interference gases, with an effective working temperature range from 25 °C to 75 °C, demonstrating the great potential of the oxygen-deficient Nb_2_O_5_ nanobelts as fast and accurate hydrogen sensing element for practical hydrogen leak detection applications.

## Data availability

The data of XRD, *I*–*V* characteristics, SEM images and sensor testing results to support the article is included in the ESI.†

## Author contributions

P. Y: investigation, formal analysis, visualization, writing – original draft, funding acquisition. Q. G.: investigation, formal analysis. Y. F.: validation, visualization. C. L.: resources. S. L.: project administration. Y. Z.: conceptualization, funding acquisition. X. Z.: writing – review & editing, methodology H. G.: funding acquisition, supervision. Z. W.: funding acquisition, resources.

## Conflicts of interest

There are no conflicts to declare.

## Supplementary Material

RA-015-D4RA08878F-s001
