# Supplementary material for: Fast and selective room-temperature hydrogen sensing of oxygen-deficient orthorhombic Nb2O5 nanobelts
Source: RSC Adv. 2025 Apr 22;15(16):12622–8. doi: 10.1039/d4ra08878f (PMC12012606; doi:10.1039/d4ra08878f)
Supplement: RA-015-D4RA08878F-s001 [file RA-015-D4RA08878F-s001.pdf]

Supplementary Information (SI) for RSC Advances.
This journal is © The Royal Society of Chemistry 2025

Supplementary Information

Fast and Selective Room-Temperature Hydrogen Sensing of Oxygen-Deficient Orthorhombic Nb_2O_5 Nanobelts

Piaoyun Yang,[1,†] Qinyuan Gao,[2] Yijing Fan,[1] Chunya Luo,[1] Sha Li,[1] Yanan Zou,[3,†] Xianghui Zhang,[2, †] Haoshuang Gu[2] and Zhao Wang[2]

[1]Hubei Expert Workstation of Terahertz Technology and Advanced Energy Materials & Devices, College of Physics and Electromechanical Engineering, Hubei University of Education, Wuhan 430205, P.R. China.

[2]Hubei Key Laboratory of Micro/Nano-Electronic Materials and Devices, School of Microelectronics, Hubei University, Wuhan 430062, P.R. China

[3]College of Science, Jilin Institute of Chemical Technology, Jilin 132000, P.R. China

† Corresponding authors, e-mail address: yangpiaoyun@hue.edu.cn (P. Yang), xhzhang@hubu.edu.cn (X. Zhang), zouyanan@jlict.edu.cn (Y. Zou)

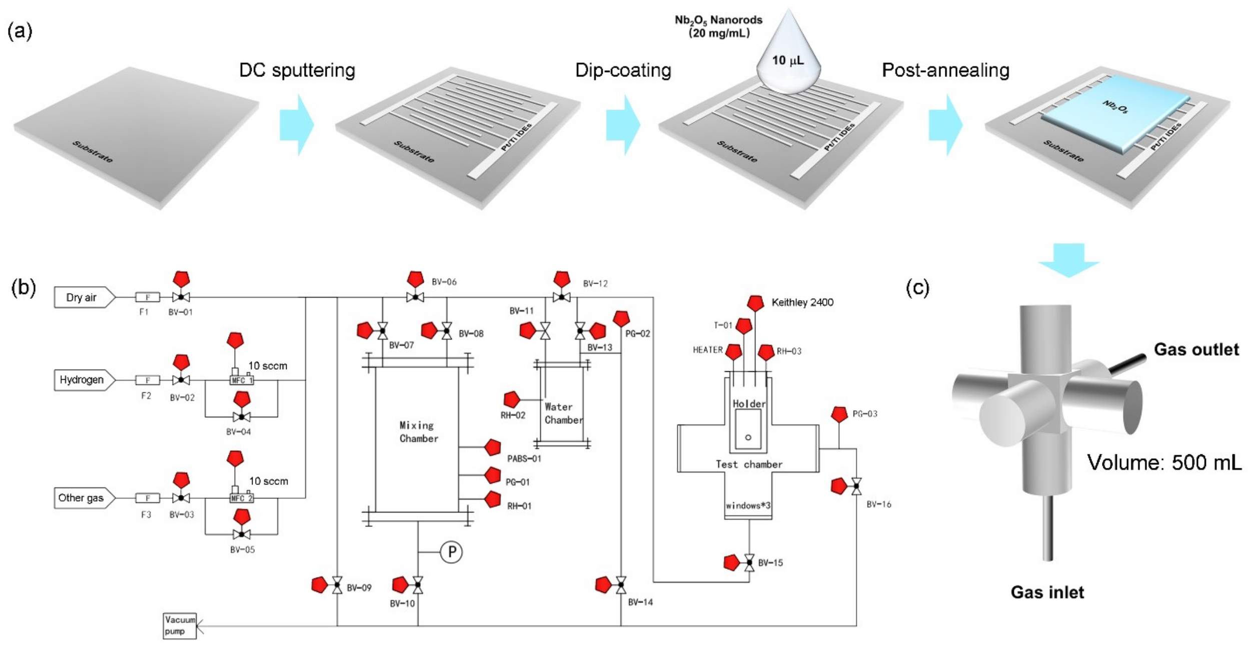

Figure S1. The schematic diagrams for the preparation of Nb_2O_5-based hydrogen sensors and the structural diagram of the gas sensing testing system. (a) Fabrication procedure for hydrogen sensors. (b) Structural diagram of the sensor testing system, wherein BV, MFC and F, PABS, PG, RH, and T represents the electromagnetic valve, mass flow controller, gas check valve, Vacuum gauge, pressure gauge, humidity sensor and temperature sensor, respectively. (c) Schematic diagram of the testing chamber.

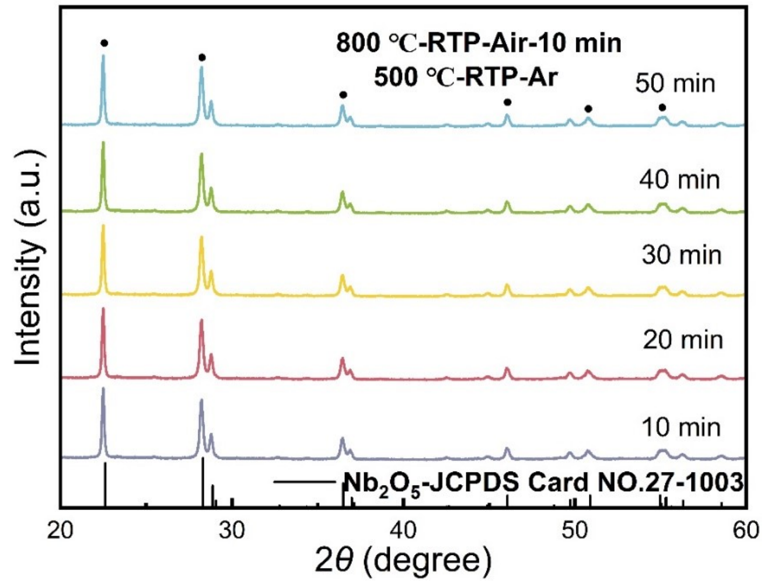

Figure S2. The XRD curves of the orthorhombic Nb_2O_5 nanobelts obtained after post annealing treatment.

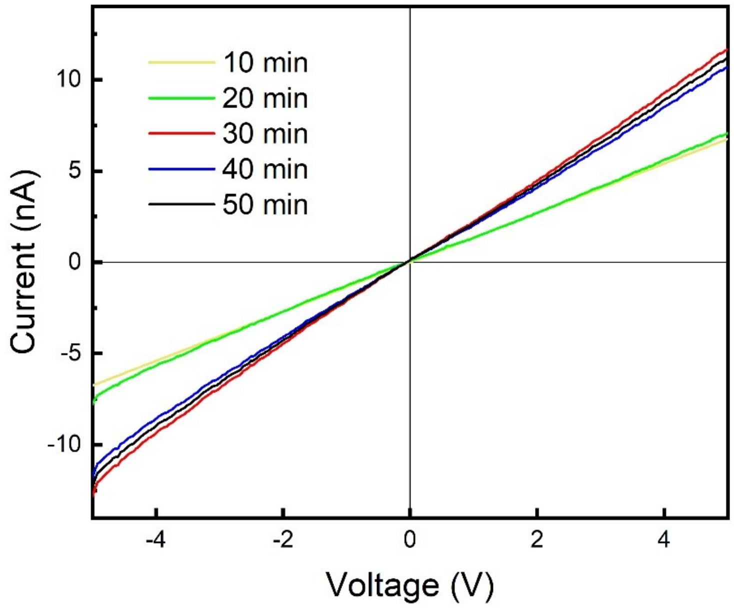

Figure S3. *I-V* characteristics of chemiresistive sensors based on the post-annealed Nb_2O_5 nanobelts, measured at room temperature in air. Samples with post-annealing duration of 10 and 20 min show much higher resistance than that of samples with longer post-annealing time. Regarding the modulation of oxygen vacancies and the polycrystalline interfaces, the resistance change between 10/20-min and 30/40/50-min groups in *I-V* characteristics mainly stems from two competing mechanisms: (1) The increased oxygen vacancies during post-annealing process generally enhance conductivity; (2) Simultaneously generated nanojunctions and grain boundaries through fragmentation and polycrystallization act as electron scattering centers, leading to overall resistance elevation.

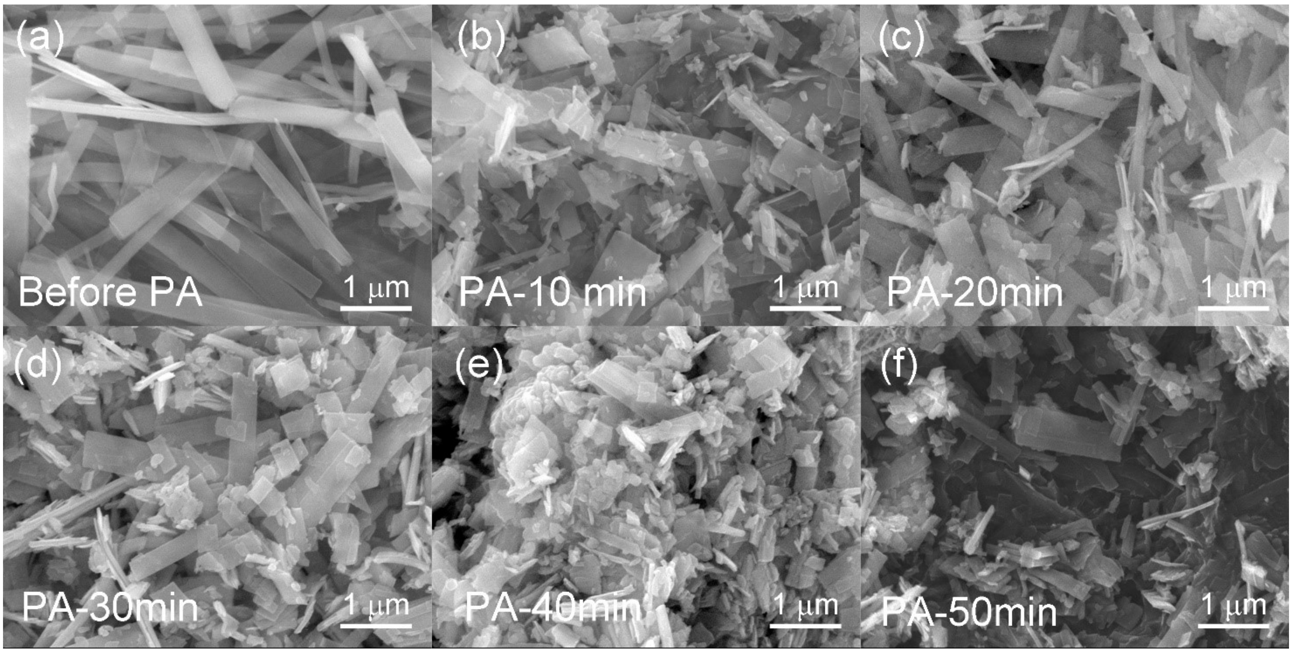

Figure S4. The SEM images of as-prepared Nb_2O_5 nanobelts before and after the post annealing treatment for different durations. (a) before post annealing; (b) 10 min; (c) 20 min; (d) 30 min; (e) 40 min; (f) 50 min.

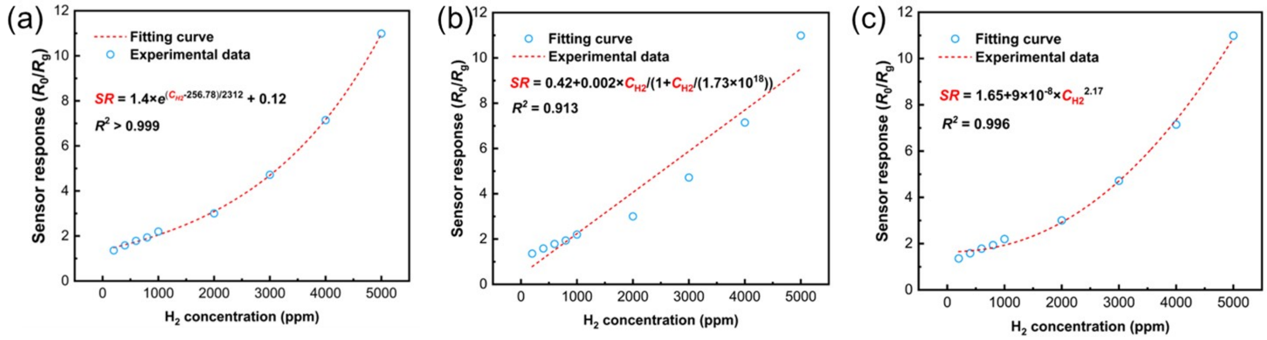

Figure S5. The non-linear fitting to the sensor response curves using (a) exponential model, (b) Langmuir model with $R_0/R_g \propto C_{H2}/(1+C_{H2}/C_{sat})$, and (c) Power-law model with $R_0/R_g \propto C_{H2}{}^{n}$, respectively. Either the Langmuir model or the Power-law model cannot well describe the concentration-dependent sensor response (e.g. the Power-law model shows clear deviation at low concentration region), while the exponential model proposed in this work according to the thermionic emission-dominated transport model can well describe the experimental data at the full detection range. Therefore, we can draw the conclusion that while Langmuir/power-law models are widely used for adsorption-dominated systems, the interfacial barrier modulation in our Nb_2O_5 nanobelt network necessitates an exponential response model.

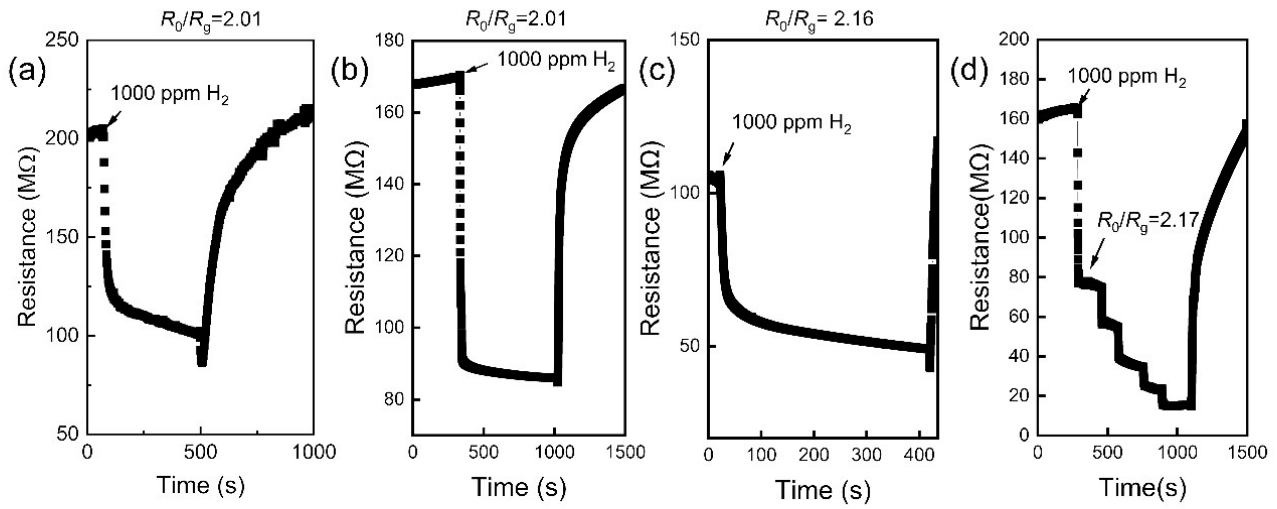

Figure S6. Room-temperature chemiresistive hydrogen response of 4 different samples to 1000 ppm hydrogen in air. Across 4 independent samples, the sensor response to 1000 ppm hydrogen gas is 2.01, 2.01, 2.16, and 2.17, respectively, which shows an error range of ±8%, confirming the good reproducibility of the hydrogen sensing performance. Moreover, baseline resistance exhibits larger deviation from ~105 to 205 MW, which shows an error range of about ±60%. The variability can be attributed to the inconsistencies in nanobelt dispersion, annealing temperature gradients, and ambient humidity during device assembly, etc.

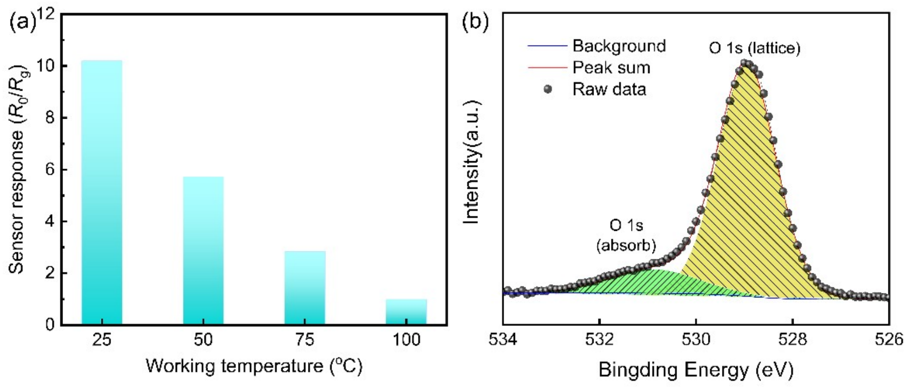

Figure S7. (a) The variation of sensor response with increasing working temperature and (b) the XPS O 1s spectrum of the Nb_2O_5 nanorods treated at 100 °C in air, showing significantly decreased content of the adsorbed oxygen species.

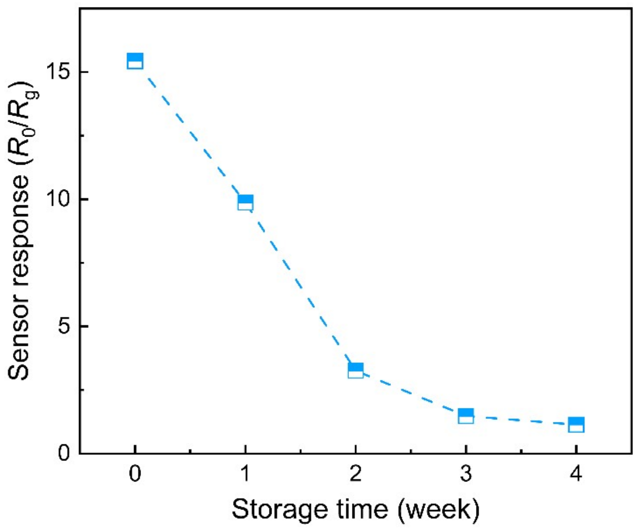

Figure S8. The room-temperature sensor response to 5000 ppm hydrogen gas at room temperature during long-term storage process.
